# Sex-specific ultrasound imaging biomarkers of neurodegeneration in a mouse model

**DOI:** 10.3389/fnagi.2024.1445164

**Published:** 2025-01-07

**Authors:** Mark B. Russell, Drew P. Locke, Haley M. Adams, Alexander R. Pelley, Rojan Saghian, Alexandre S. Maekawa, Darcie Stapleton, Grace V. Mercer, John G. Sled, Lindsay S. Cahill

**Affiliations:** ^1^Department of Chemistry, Memorial University of Newfoundland, St. John’s, NL, Canada; ^2^Mouse Imaging Centre, Hospital for Sick Children, Toronto, ON, Canada; ^3^Translational Medicine, Hospital for Sick Children, Toronto, ON, Canada; ^4^Department of Medical Biophysics, University of Toronto, Toronto, ON, Canada; ^5^Discipline of Radiology, Memorial University of Newfoundland, St. John’s, NL, Canada

**Keywords:** cerebrovasculature, mouse, neurodegeneration, pulse wave velocity, sex differences, ultrasound

## Abstract

**Introduction:**

Early detection of neurodegeneration is essential for optimizing interventions. The highly reproducible progression of neurodegeneration in the decrepit (*dcr*) mouse allows investigation of early biomarkers and mechanisms of brain injury.

**Methods:**

Using high-frequency ultrasound, the common carotid arteries of female and male *dcr* and control mice were imaged longitudinally at time points bracketing the disease progression (50, 75, and 125 days of age) (*n* = 6 mice/group/sex).

**Results:**

Over the disease time course, the female *dcr* mice demonstrated increased carotid artery blood flow and pulse wave velocity while the male *dcr* mice had a decrease in heart rate and no change in carotid artery ultrasound parameters. Early imaging biomarkers were sex-specific, with decreased carotid artery blood flow in female *dcr* mice and increased carotid artery diameter and decreased pulse wave velocity in males.

**Discussion:**

Carotid artery and wave reflection ultrasound is a promising screening tool for early detection of neurodegeneration.

## 1 Introduction

Biomedical imaging has resulted in significant improvements in the diagnosis of neurodegeneration. Imaging biomarkers that detect pathology before the emergence of overt clinical symptoms are critical to enable early treatment interventions. One promising non-invasive imaging modality for physiological biomarker detection is ultrasound. Cerebral hypoperfusion measured using transcranial Doppler ultrasound has been associated with dementia and may precede the onset of clinical neurodegeneration (Ruitenberg et al., 2005). Arterial vessel stiffness, determined by carotid-femoral pulse wave velocity (PWV) ultrasound measurements, has also been associated with neurodegenerative disease (Hanon et al., 2005; Pase et al., 2016; Rouch et al., 2018). While the use of ultrasound shows promise for the early detection of neurodegeneration, several studies have found no association with ultrasound-derived parameters and neurodegeneration (Sugawara et al., 2010; Nilsson et al., 2017). These differences may be attributed to differences in study population including age, genetic factors, biological sex, and comorbidities. Experimental animal models play an important role in biomarker discovery, with high genetic similarity and strict control of environmental factors such as diet and housing.

Our group has described a novel mouse model, termed “decrepit” (*dcr*), that arose from a spontaneous mutation in a mitochondrial-associated gene (mitochondrial ribosomal protein L3, *Mrpl3*) in a C57BL6/J mouse colony (Sproule et al., 2010). The phenotype of these mice includes adult-onset neurodegeneration, atrophy in the hippocampus, a progressive memory impairment, and a shortened lifespan (Cahill et al., 2020). Disease onset occurs at 70 days of age and premature death occurs by 150 days of age. The progressive degeneration in the *dcr* mice is highly reproducible and follows a neurological pathway that is implicated in Alzheimer’s disease (Apostolova et al., 2007), making this mouse model ideal for the study of biomarkers in neurodegeneration.

In the present study, we used high-frequency ultrasound and a longitudinal study design to measure changes in carotid artery blood flow in female and male *dcr* mice compared to healthy controls. In addition, we used a methodology developed by our group, termed wave reflection ultrasound, to study pulse pressure waves as they move along the carotid artery (Macgowan et al., 2015). This methodology has demonstrated sensitivity to cerebrovascular changes in a genetic model of sickle cell disease (Cahill et al., 2019) and in mice exposed to traumatic brain injury (Steinman et al., 2020). In addition, because the incidence and progression of neurodegeneraton is known to be dependent on biological sex in humans (Gambassi et al., 1999; Larson et al., 2004; Beam et al., 2018), we assessed whether the ultrasound parameters showed any sex differences.

## 2 Materials and methods

### 2.1 Animals

The *dcr* mouse model has an insertion of at least 133 bp within introns 6-7 of Mrpl3 (mitochondrial ribosomal protein L3) (Sproule et al., 2010; Cahill et al., 2020). The *dcr* mice were rederived at The Centre for Phenogenomics (TCP) and bred in-house at the Health Sciences Animal Facility at Memorial University of Newfoundland. Twelve homozygous *dcr* mice (six females and six males) and twelve wild-type controls (without the *dcr* mutation) (six females and six males) were used in the study. Mice were co-housed in a standard cage on a 12 h light:dark cycle, with *ad libitum* access to food and water. All animal experiments were approved by the Animal Care Committee at Memorial University of Newfoundland and conducted in accordance with the ARRIVE guidelines and guidelines established by the Canadian Council on Animal Care.

### 2.2 Genotyping

DNA was extracted from frozen tail samples using an alkaline lysis method. Each sample was treated with 250 μL of 50 mM NaOH. The samples were vortexed to ensure complete immersion of the tail tissue in the alkaline solution. The samples were then incubated at 98°C for 30 min to facilitate DNA release. Following incubation, the samples were vortexed vigorously to further aid in DNA extraction. The samples were centrifuged at 15,000 rcf for 5 min at room temperature to pellet insoluble debris. From the resulting supernatant, 20 μL was transferred to a new tube and diluted with 80 μL of TE buffer (10 mM Tris-HCl, 1 mM disodium EDTA, pH 8.0). A total of 2 μL of the diluted DNA template was either utilized for PCR analysis or stored at −20°C for future use.

Polymerase chain reaction (PCR) was performed using NEB Taq Polymerase (New England Biolabs, Whitby, ON, Canada). The PCR reaction mix contained 13.4 μL of nuclease-free water, 2 μL of buffer (10 mM Tris-HCl, 50 mM KCl, 1.5 mM MgCl_2_, pH 8.3), 1.2 μL of MgCl_2_, 0.4 μL of dNTPs, 0.4 μL of each primer, and 0.2 μL of Taq polymerase. The primers used for amplification were Jax-F (TCT CGA GAG TCA GCT CAT AGA GAC A), Insertion-F (CAG GAA CAC CTC GAT GCT C), and Jax-R (CCC CGC AGA GAC TCA TTA CCT). The PCR cycling conditions consisted of an initial denaturation at 95°C for 3 min, followed by 35 cycles of 95°C for 30 s, 58°C for 15 s, and 72°C for 1 min, with a final hold at 4°C. The PCR products were analyzed on agarose gels. Knockout (KO) mice were expected to yield two bands at 214 and ∼600 bp. Heterozygous (Het) mice were expected to yield three bands at 214, 451, and ∼600 bp, while wild-type (WT) control mice were expected to produce a single band at 451 bp.

### 2.3 Experimental design

The mice were weighed and imaged longitudinally using a high-frequency ultrasound system at three time points. The time points were chosen to bracket the disease progression (Cahill et al., 2020): 50 days of age (prior to onset of neurodegeneration), 75 days of age (first signs of overt behavioral phenotype and neurodegeneration), and 125 days (late stages of neurodegeneration, premature death).

### 2.4 Ultrasound biomicroscopy

The left and right common carotid arteries (LCCA and RCCA, respectively) were imaged using a high-frequency ultrasound system and a UHF57x transducer (centre frequency of 40 MHz) (F2, VisualSonics, Toronto, Ontario, Canada) as described previously (Macgowan et al., 2015; Cahill et al., 2019). Briefly, mice were anesthetized with isoflurane (4% for induction and 1.0–2.0% for maintenance in 100% O_2_). Body temperature was maintained at 36–37°C using a heated platform and temperature, respiration rate and heart rate was monitored throughout the experiment. These parameters were chosen based on best practices for measurements of physiology in mice (Lindsey et al., 2018). M-mode traces were recorded approximately 3 mm proximal to the carotid artery bifurcation with the transducer parallel to the arteries. Pulsed Doppler velocity spectra were recorded at the same position with the transducer at an angle of insonation less than 60°.

### 2.5 Ultrasound image analysis

All ultrasound images were analyzed as described previously (Macgowan et al., 2015; Cahill et al., 2019). The near and far vessel walls in the M-mode traces were automatically outlined, divided into cardiac cycles based on the start of systole, and averaged together. The maximum envelope of the Doppler velocity traces were traced and the final instantaneous average velocity was determined by dividing the traced envelope by two. The velocity waveforms were separated into cardiac cycles, temporally aligned and averaged together. The average area and velocity waveforms were used to calculate the average flow waveforms. The PWV was calculated by plotting flow versus area (QA loop) and fitting a line to 20–80% of the maximum systolic flow region.

The observed waveform was decomposed into its forward and reflected components as described in Cahill et al., 2019. The reflection coefficient was computed as the magnitude of the ratio of the first harmonics of the forward and reflected waveforms. The carotid artery pulsatility index was calculated as the difference between peak systolic and end diastolic velocities, divided by the mean velocity over the cardiac cycle. Young’s modulus of elasticity (*E*) was calculated using the Moens-Korteweg equation (Milnor, 1982) and 1.05 g/mL as the density of the blood (Burstain et al., 1994).

### 2.6 Statistical analysis

All statistical tests were performed using the R statistical software package.^1^ Data are presented as mean ± standard error of the mean. A value of *p* < 0.05 was defined as statistically significant. The body weight was analyzed using a linear mixed effects model with genotype (control, *dcr*), age and biological sex as fixed effects and mouse ID as the random effect to account for repeated measures. The carotid artery diameter, blood flow, PWV, pulsatility index, wall thickness, Young’s modulus, reflection coefficient, and mouse heart rate were analyzed using a linear mixed effects model with genotype (control, *dcr*), age, biological sex (male, female) and side (LCCA, RCCA) as fixed effects and mouse ID as a random effect. If the linear mixed effects model was significant, *post hoc* tests were performed by releveling the model. For parameters that were significantly different between genotypes prior to the onset of disease (50 days of age), receiver operating characteristic (ROC) curves (Hanley and McNeil, 1982) were computed to evaluate how well these parameters differentiated between the control and *dcr* mice. The area under the ROC curves (AUC) was calculated using the pROC package in R (Robin et al., 2011) and the optimal cut-off for discrimination was determined using the Youden index (Youden, 1950).

## 3 Results

**FIGURE 1:**
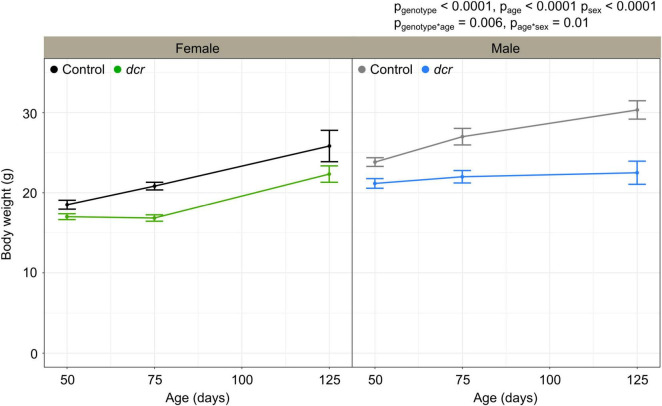
Change in body weight for female control (black), female *dcr* (green), male control (gray) and male *dcr* (blue) mice from 50 to 150 days of age. Data presented as means ± standard error of the mean. Main effects of genotype, age and sex are noted as p_genotype_, p_age_ and p_sex_ and the age-by-genotype and sex-by-age interactions as p_genotype*age_ and p_age*sex_. *n* = 6 mice/genotype/sex.

Two of the six male *dcr* mice reached endpoint (lethargy, decrease in weight >20%) before the final imaging time point and had to be euthanized. Six of the *dcr* mice (three females and three males) showed evidence of heart arrhythmias during the ultrasound scan. Figure 1 shows the change in body weight for the female and male *dcr* and control mice from 50 to 125 days of age. For the body weights, there were significant main effects of genotype (*p* < 0.0001), age (*p* < 0.0001) and sex (*p* < 0.0001). A significant age-by-genotype interaction (*p* = 0.006) demonstrated that the body weights had an altered trajectory in the *dcr* mice compared to the controls. The difference in the rate of change between the *dcr* and control mice was dependent on sex (*p* = 0.01), with *post hoc* analysis showing a significant increase in weight for the female *dcr* mice with age (*p* < 0.0001) and no change in weight for the male *dcr* mice with age (*p* = 0.4). There were no significant differences in weight between the *dcr* and controls at the first imaging time point (*p* = 0.1) and only the weight of the male *dcr* mice was different than controls at the final imaging point (*p* = 0.003).

**FIGURE 2:**
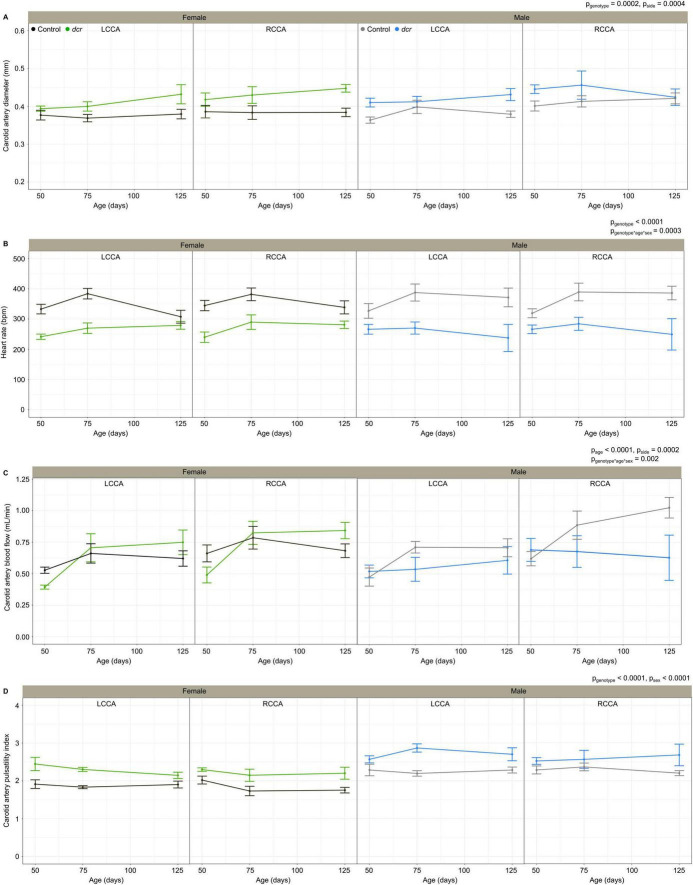
Change in carotid artery diameter **(A)**, heart rate **(B)**, carotid artery blood flow **(C)**, carotid artery pulsatility index **(D)** for female control (black), female *dcr* (green), male control (gray) and male *dcr* (blue) mice from 50 to 150 days of age for the left common carotid artery (LCCA) and the right common carotid artery (RCCA). Data presented as means ± standard error of the mean. Main effects of genotype, age, sex and side are noted as p_genotype_, p_age_, p_sex_ and p_side_ and the sex-by-age-genotype interaction as p_genotype*age*sex_. *n* = 6 mice/genotype/sex.

Figure 2 shows the longitudinal ultrasound measurements for the LCCA and RCCA of the female and male *dcr* and control mice. As we have reported previously (Cahill et al., 2019), the carotid artery diameter and blood flow were significantly larger for the RCCA compared to the LCCA (*p* = 0.0004 and *p* = 0.0002 respectively). The carotid artery diameter (Figure 2A) was significantly larger in the *dcr* mice (*p* = 0.0002) and the heart rate (Figure 2B) was significantly lower in the *dcr* mice compared to controls (*p* < 0.0001). *Post hoc* analysis at the first imaging time point showed there was no difference in the carotid artery diameter between the female *dcr* mice and the controls (*p* = 0.2), while the male *dcr* mice had a 12% larger carotid artery diameter than the controls (*p* = 0.009). This is in contrast to the final imaging time point, where the carotid artery of the female *dcr* mice was now 15% larger than the controls (*p* = 0.01) and there was no difference between *dcr* and control males (*p* = 0.2). How the heart rate in the *dcr* and control mice changed with age was dependent on sex (*p* = 0.0003). *Post hoc* analysis showed the male *dcr* mice have a decrease in heart rate with increasing age compared to controls (*p* = 0.04) and while the heart rate of female *dcr* mice was 29% lower at the first imaging time point (*p* = 0.001), it was the same as controls at the final time point (*p* = 0.1).

The carotid artery blood flow (Figure 2C) did not differ based on genotype (*p* = 0.1); however, blood flow increased significantly with age (*p* = 0.0001) and the change in blood flow with age for the *dcr* and control mice was different between males and females (*p* = 0.002). *Post hoc* analysis showed the female *dcr* mice had a 25% lower carotid artery blood flow than controls at the first imaging time point (*p* = 0.02), increasing to the same blood flow as the controls by the second imaging time point (*p* = 0.7) and a trend toward an increase in blood flow compared to controls at the final imaging time point (*p* = 0.1). For the male *dcr* mice, the opposite was observed with no difference in carotid artery blood flow at the first imaging time point (*p* = 0.5) and no increase with age (*p* = 0.9), resulting in a trend toward lower blood flow compared to controls at the last imaging time point (*p* = 0.1).

The carotid artery pulsatility index (Figure 2D), a surrogate measure of vascular resistance, was significantly larger in the *dcr* mice compared to controls (*p* < 0.0001) and was different between females and males (*p* < 0.0001). *Post hoc* tests showed the pulsatility index was larger in *dcr* females compared to controls at all three imaging time points (*p* < 0.01) and only larger in *dcr* males compared to controls during the second and third imaging time points (*p* < 0.04).

**FIGURE 3:**
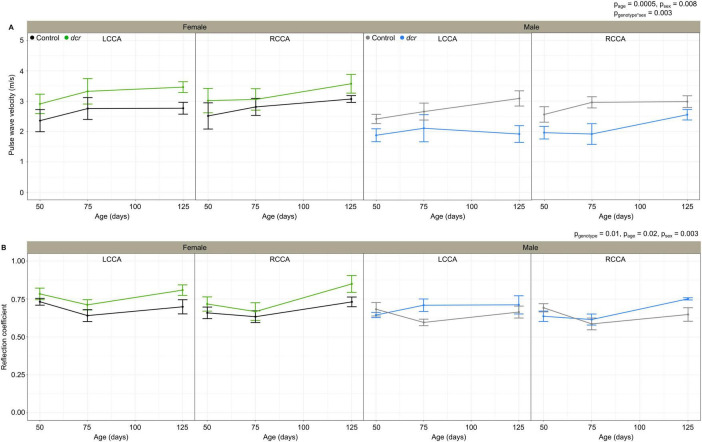
Change in carotid artery pulse wave velocity **(A)** and reflection coefficient **(B)** for female control (black), female *dcr* (green), male control (gray) and male *dcr* (blue) mice from 50 to 150 days of age for the left common carotid artery (LCCA) and the right common carotid artery (RCCA). Data presented as means ± standard error of the mean. Main effects of genotype, age and sex are noted as p_genotype_, p_age_ and p_sex_ and the sex-by-genotype interaction as p_genotype*sex_. *n* = 6 mice/genotype/sex.

**FIGURE 4:**
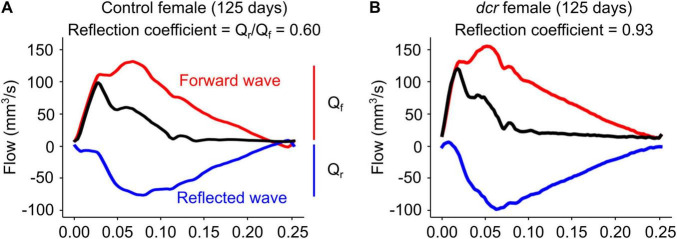
Representative examples of decomposed forward (red, Q_f_) and reflected (blue, Q_r_) waves from the observed common carotid artery waveform (black) at 125 days of age in panel **(A)** control female and **(B)** *dcr* female mice. The reflection coefficient was computed as the magnitude of the ratio of the first harmonics of the forward and reflected waveforms.

The PWV (Figure 3A), a marker of arterial stiffness, was significantly dependent on age (*p* = 0.0005) and sex (*p* = 0.008) and there was a significant sex-by-genotype interaction (*p* = 0.003). *Post hoc* analysis showed a 22 and 30% decrease in PWV at the first (*p* = 0.04) and final imaging time point (*p* = 0.01) for the male *dcr* mice compared to controls. In contrast, the PWV for the *dcr* female mice was increased by 21% compared to controls at the final imaging time point (*p* = 0.04). The reflection coefficient (Figure 3B) was significantly dependent on genotype (*p* = 0.01), age (*p* = 0.02) and sex (*p* = 0.003), with *post hoc* tests showing only the female *dcr* mice had a statistically elevated reflection coefficient at the final imaging time point (*p* = 0.02). Figure 4 includes representative examples of the decomposed forward and reflected pulse pressure waves in female mice at 125 days of age. The carotid artery wall thickness was increased in the *dcr* mice compared to controls (*p* = 0.008). Similar to the PWV, the Young’s modulus of elasticity was dependent on age (*p* = 0.0009), sex (*p* = 0.02) and had a sex-by-genotype interaction (*p* = 0.01). The Young’s modulus for the female *dcr* mice is 28% larger than controls by the final imaging time point.

Several ultrasound parameters were significantly different between the control and *dcr* mice at the first imaging time point, before the onset of neurodegeneration. For the females, ROC analysis for the ability of the carotid artery blood flow to discriminate between control and *dcr* mice gave an AUC value of 0.79. The point on the ROC curve that optimizes sensitivity and specificity is a carotid artery blood flow value of 0.49 mL/min. For the males, the AUC values for the carotid artery diameter and the PWV to discriminate the genotypes were 0.83 and 0.76, respectively and the optimal cut-off points were 0.41 mm and 2.62 m/s, respectively.

## 4 Discussion

Using longitudinal high-frequency ultrasound imaging, this study demonstrated altered heart rate, carotid artery diameter and wall thickness, and cerebrovascular resistance in a mouse model of neurodegeneration. The decreased heart rate and evidence of heart arrhythmias in the *dcr* mice suggest previously undocumented cardiac dysfunction. Several studies have reported links between neurodegeneration and decreased heart rate (Bayón et al., 2014) and atrial fibrillation (Madhavan et al., 2018; Liu et al., 2022). A detailed evaluation of cardiac function and the heart-brain axis in the *dcr* mice will be the subject of future investigations. The *dcr* mice may represent a new model of cardiovascular disease. The observed increase in carotid artery diameter and wall thickness match clinical findings in dementia (Newman et al., 2005; van Oijen et al., 2007; Gutierrez et al., 2016; Wang et al., 2021) and suggest a cerebrovascular adaptation to maintain blood flow to the brain. Previous work using X-ray micro-computed tomography imaging of the cerebrovasculature in *dcr* mice showed significant vascular proliferation in response to the neurodegeneration (>100 days of age) (Cahill et al., 2020). This is consistent with the abnormal reflection coefficient in the *dcr* mice that was more pronounced at the final imaging time point. An increased reflection coefficient has also been reported in other studies of altered cerebrovascular morphology such as exposure to acute hypercapnia (Macgowan et al., 2015) and in a mouse model of sickle cell disease (Cahill et al., 2019). The reflection coefficient isolates the component of the Doppler signal that is specific to the cerebral vasculature and is therefore a sensitive measure of vascular resistance and geometry (Sled et al., 2019; Cahill et al., 2019). In contrast, the pulsatility index is dependent on multiple hemodynamic variables in addition to vascular resistance, including heart rate and perfusion pressure (de Riva et al., 2012). There was a strong negative correlation between the pulsatility index and heart rate (*p* = 0.0004) and therefore the observed decreased heart rate in the *dcr* mice likely explains the elevated pulsatility index.

An interesting feature of this study was the striking biological sex differences. While Sproule et al. (2010) reported that the genetic mutation had a similar effect on the behavior of both female and male *dcr* mice (hyperexcitability, followed by wasting, lethargy and premature death), body weight and histological analyses were only performed on males. In addition, when sex was included as a covariate in the magnetic resonance imaging analysis there was no difference in the altered trajectory of brain structure volumes between female and male *dcr* mice from 50 to 150 days (Cahill et al., 2020). In the present study, all of the experimental assays showed biological sex differences. The female *dcr* mice had an initial growth deficit relative to controls followed by growth at a normal rate while the male *dcr* mice had a progressive growth deficit throughout their lifetime. While the ultrasound parameters in the female *dcr* mice increased with age, there was no change with age for the male *dcr* mice. Specifically, the female *dcr* mice have the ability to increase blood flow (and delivery of oxygen and nutrients) to the brain as neurodegeneration progresses, whereas the male *dcr* mice showed no changes in blood flow. This hypoperfusion observed in the male *dcr* mice is one of the hallmarks of neurodegeneration and may contribute to the development of dementia (Ruitenberg et al., 2005). The ability to increase carotid artery blood flow with age may explain the improved outcomes for the females compared to the males, with mortality within the timeframe of the study only observed for *dcr* male mice (*n* = 2). These observed biological sex differences in mortality are similar to Alzheimer’s disease in humans, as women tend to live longer than men following diagnosis (Gambassi et al., 1999; Larson et al., 2004). Future studies will investigate the impact of sex chromosomes and sex hormones on these differences (e.g., using an ovariectomized mouse model).

The *dcr* mice showed evidence of abnormal PWV, with the effect depending on the biological sex. While both females and males show evidence of altered mechanical properties of the vessel walls, the increased PWV in the female *dcr* mice suggests decreased vascular compliance specific to the female mice. To our knowledge, sex differences in arterial stiffness have not been explored in a human population with neurodegenerative diseases. However, arterial stiffness in humans is known to increase with normal, healthy aging in both biological sexes (Kohn et al., 2015), and is associated with neuroinflammation, synaptic dysfunction, phosphorylated tau, and total tau in older adults (Hughes et al., 2014; Moore et al., 2021). PWV is also elevated in cases of mild cognitive impairment, vascular dementia and Alzheimer’s disease compared to those without cognitive impairment (Hanon et al., 2005; Pase et al., 2016; Rouch et al., 2018; Wang et al., 2021). Arterial stiffness is higher in healthy older women compared to men (Waddell et al., 2001; Noon et al., 2008) and is associated with higher mortality from cardiovascular disease in older women than in men (Regnault et al., 2012; Coutinho, 2014). The mechanisms behind these biological sex differences are still being investigated but a recent review details the possible impact of hormonal status, diet and exercise (DuPont et al., 2019). The history of pregnancy and childbirth may also play a role in neurovascular aging (Miller et al., 2020).

At the first imaging timepoint in this study (50 days of age), the *dcr* mice demonstrate no behavioral symptoms, cognitive deficits or any evidence of neurodegeneration (from magnetic resonance imaging or immunohistochemistry) (Sproule et al., 2010; Cahill et al., 2020). Despite this, several imaging parameters were different between controls and *dcr* mice at 50 days of age that were dependent on biological sex. For the female mice, the carotid artery blood flow was significantly lower in the *dcr* mice compared to controls at the first imaging time point. This is consistent with Ruitenberg et al. (2005) where cerebral hypoperfusion was associated with subsequent dementia. For the male mice, the carotid artery diameter was significantly larger and the PWV was lower in the *dcr* mice compared to controls. A recent longitudinal population-based study showed adults with increased carotid artery diameter on magnetic resonance imaging were more likely to develop dementia later in life (Melgarejo et al., 2024). The reduction in the PWV in the male *dcr* mice was surprising; however, it may be explained by a significant decrease in arterial blood pressure. In both females and males, these early vascular adaptations are likely a compensatory response to provide the brain with additional oxygen. However, as observed in the *dcr* male mice, if the carotid artery is maximally dilated early in the disease, it cannot continue to adapt and it is more susceptible to brain tissue damage from environmental stressors because of the inability to autoregulate. Anecdotally, the two male *dcr* mice that reached their endpoint before the final imaging time point had the largest carotid artery diameters even at 50 days (17% larger than male controls and 7% larger than the other male *dcr* mice who survived). Additionally, both the heart rate (29% lower than male controls and 20% lower than other male *dcr* mice who survived) and the PWV (43% lower than male controls and 36% lower than other male *dcr* mice who survived) were lowest in these two mice even at 50 days of age. The predictive performance of these imaging biomarkers was moderate, with the carotid artery diameter in males showing the highest AUC value of 0.83. These results support further study of potential early biomarkers of neurodegeneration in a sex-specific manner.

The timing of alterations in the ultrasound parameters provides insight into the mechanisms of the neurodegeneration in the *dcr* mice and how these differ between females and males. The increased arterial stiffness in the female *dcr* mice means the large arteries are no longer able to dampen the pulse pressure from the heart, likely resulting in damage to the cerebral microvasculature (Chirinos et al., 2019). On the other hand, the decreased carotid artery blood flow in the male *dcr* mice may cause hypoxia in vulnerable brain areas. While both female and male *dcr* mice undergo neurodegeneration along a similar pathway that results in premature death, the mechanism of the neurodegeneration may be different. Future studies will examine changes at the cellular and molecular level to further explore these sex-specific mechanisms.

The present study has several limitations. Arterial blood pressure is known to make a significant contribution to arterial stiffness (Blacher et al., 1999); however, we were unable to reliably measure blood pressure in mice. Another limitation is the requirement for isoflurane anesthesia during the ultrasound imaging. Isoflurane is known to have cardiovascular effects (e.g., tachycardia) and, while we aim to keep the level and exposure time similar between groups of mice, the absolute blood flow values will not match measurements in awake animals. A recent study reported a sex-specific effect of isoflurane in a mouse model of Alzheimer’s disease, with a decrease in the heart rate of male mice and not female mice during the first 30 min of the exposure (Bratke et al., 2024). While we did not observe this difference in heart rate change over the imaging sessions in the present study, it is important to consider the influence of anesthesia on cerebral blood flow dynamics when interpreting study findings.

## 5 Conclusion

In summary, the use of a longitudinal ultrasound study and a mouse model with highly reproducible brain pathology allowed us to identify several potential early biomarkers of neurodegenerative disease. Compared to other imaging modalities, ultrasound is non-invasive, easy to use, relatively inexpensive and accessible. As such, carotid artery ultrasound shows promise as a clinical screening tool for the early detection and monitoring of the progression of neurodegenerative diseases. Consideration of the biological sex of the patient will be critical and further research is required to establish an understanding of the impact of other patient characteristics (ethnicity/race, gender). In addition to the standard measures for ultrasound evaluation of the carotid artery (diameter, blood flow), measurements of PWV and wave reflections may also provide a novel diagnostic approach.

## Data Availability

The raw data supporting the conclusions of this article will be made available by the authors, without undue reservation.

## Footnotes

1
www.r-project.org
